# Biomechanical Specialization Acts as an Asymmetrical Constraint on the Phenotype

**DOI:** 10.1093/iob/obaf013

**Published:** 2025-04-07

**Authors:** M I Schelp, E D Burress

**Affiliations:** Department of Educational Psychology, Research Methodology, and Counseling, University of Alabama, Tuscaloosa, AL 35487, USA; Department of Biological Sciences, University of Alabama, Tuscaloosa, AL 35487, USA

## Abstract

Vertebrate jaws involve trade-offs between the transmission of velocity and force, which underlies their feeding performance and potentially their evolution. We investigate the velocity–force trade-off and its implications for adaptation of the anatomically complex fish jaw system among 89 species of percid fishes (Percidae). We test alternative hypotheses about how the trade-off may symmetrically or asymmetrically constrain jaw diversity. We find that the trade-off has a strong impact on the structural diversity of the jaws, indicating that specialization acts as a constraint on the phenotype. Force-modified jaws are compact with short snouts and a small oral cavity, while velocity-modified jaws are more robust with elongate snouts and a large oral cavity. The distribution of craniofacial diversity along the extremes is asymmetrical, as species with velocity-modified jaws are more phenotypically dissimilar than those with force-modified jaws. The rate of phenotypic evolution is also asymmetrical, as lineages with velocity- and force-modified jaws evolve slower and faster than unspecialized jaws, respectively. This discrepancy between phenotypic diversity and rate of evolution is explained by time to evolve, as force-modified jaws arose comparatively nearer the present. We expand recent literature linking trade-offs to asymmetrical macroevolutionary patterns, which may be an underappreciated cause of the uneven distribution of vertebrate diversity.

## Introduction

Trade-offs inherently impose a constraint on anatomy, life history, immunity, or other biological processes (Reznick 1983; Stearns 1989; Adamo et al. 2001; Zera and Harshman 2001; Uicker et al. 2003; Sadras 2007; Schwenke et al. 2016; Koch and Hill 2018). For example, the lever systems imbedded throughout much of vertebrate anatomy (e.g., limbs and jaws) are subject to a trade-off between the transmission of velocity and force (Biewener 1989; Westneat 1994, 2004; Patek et al. 2007; Wainwright 2007; Zelditch et al. 2017). In addition to these mechanical properties, such trade-offs have a broad influence on emergent functional and ecological properties, including locomotor and feeding performance as well as habitat and prey preferences (Losos 1990; Losos et al. 1993; Wainwright et al. 2004; Corn et al. 2021).

The velocity–force trade-off is an inherent property of vertebrate jaw systems such that the jaws cannot be modified to transmit more velocity and force (Westneat 1994, 2004; Wainwright 2007, Wainwright et al. 2007). However, it is less clear how this trade-off might influence surrounding anatomical structures such as the craniofacial system. Many different phenotypes may produce the same mechanical properties (Wainwright et al. 2005) and many groups with exceptional phenotypic diversity have thoroughly explored the velocity–force trade-off (Wainwright et al. 2004; Wainwright 2007). Many macroevolutionary studies have focused on emergent properties of trade-offs (e.g., diet or feeding modes) rather than the underlying trade-off itself, and have broadly pointed to feeding ecology having a strong effect on rates of jaw evolution (Borstein et al. 2019; Arbour et al. 2020; Corn et al. 2021, 2022; Burress et al. 2023). Similarly, the velocity–force trade-off can strongly influence the rate of evolution (Holzman et al. 2012; Burress et al. 2020; Burress and Muñoz 2023).

Possible macroevolutionary implications of trade-offs include funneling lineages into a limited number of possible phenotypes (Cooper and Westneat 2009; Burress and Muñoz 2023) and/or acting as a catalyst/constraint on rates of phenotypic evolution (Holzman et al. 2012; Burress et al. 2023; Burress and Muñoz 2023). Traits engaged in trade-offs may evolve rapidly (Holzman et al. 2012) and specialization along the extremes of a trade-off may promote rapid phenotypic evolution (Burress and Muñoz 2023), although the underlying trade-off inherently constrains the phenotype (Westneat 1994, 2004). Thus, trade-offs may have complex, often opposing, mechanical and macroevolutionary implications.

Fish jaws are structurally complex and comprised of many lever systems (Westneat 1994) that underlie their functionality and ultimately, their emergent feeding ecology (Martinez et al. 2018; Burress et al. 2023). Previous work found that specialization along the extremes of the velocity–force trade-off led to rapid evolution of the surrounding jaw system in cichlid fishes, despite acting as a constraint on jaw diversity (Burress and Muñoz 2023). Cichlids have highly kinetic oral jaws (Waltzek and Wainwright 2003; Martinez et al. 2018; Burress et al. 2020) that are structurally and functionally reminiscent of most spiny-rayed fishes (Bellwood et al. 2015; McGee et al. 2016), but have exceptionally versatile pharyngeal jaws that may ease functional drawbacks associated with highly specialized oral jaws (i.e., Liem 1973). Therefore, it remains unclear whether specialization along the extremes of the velocity–force trade-off acting as a catalyst of jaw evolution is a general feature of fish jaws or a more limited phenomenon enabled by highly mobile and modified oral and pharyngeal jaws, respectively.

In many darters and their allies (Percidae), the premaxilla is fixed such that the ascending process does not slide along the nasal bone, resulting in minimal jaw protrusion (Carlson and Wainwright 2010). Since suction feeding is normally achieved via a combination of lower jaw rotation, cranial rotation, buccal depression, and jaw protrusion (Martinez et al. 2018; Corn et al. 2021), darters likely disproportionately rely upon the former three motions. Further, darters have simple pharyngeal jaws, notably lacking the modified anatomy of cichlids (Wainwright et al. 2012). Therefore, darters have comparatively less dynamic oral and pharyngeal jaw systems than cichlids and may provide further insight into how the velocity–force trade-off influences the diversity of the head and jaws.

**Fig. 1.**
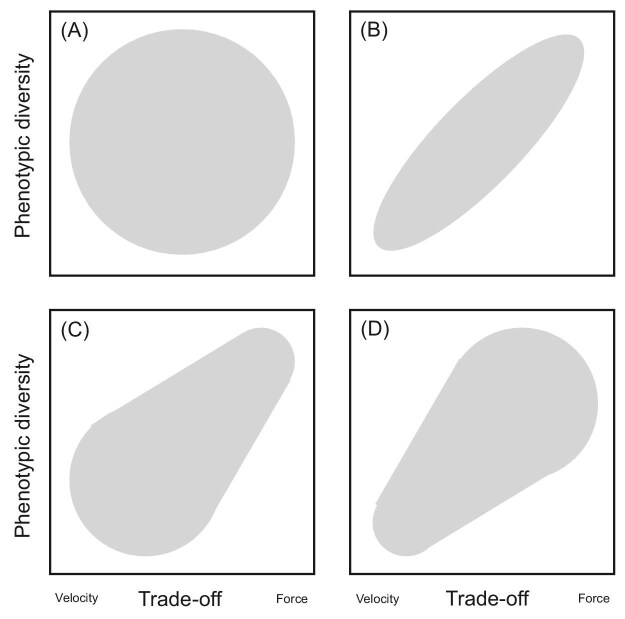
Hypothetical phenotypic diversity as a function of the velocity–force trade-off: the trade-off does not act as a constraint on phenotypic diversity (**A**), acts as a symmetrical constraint on phenotypic diversity (**B**), acts as an asymmetrical constraint in which force-modified jaws are disproportionately constrained (**C**), and acts as an asymmetrical constraint in which velocity-modified jaws are disproportionately constrained (**D**).

In this study, we assessed the role of specialization along the velocity–force trade-off as a constraint on craniofacial diversity and the rate of jaw evolution in percid fishes. We tested four possible relationships between craniofacial diversity as a function of the velocity–force trade-off: specialization (1) does not act as a constraint on the phenotype, (2) acts as a symmetrical constraint, (3) acts as an asymmetrical constraint in which velocity-modified jaws are disproportionately constrained, and (4) acts as an asymmetrical constraint in which force-modified jaws are disproportionately constrained (Fig. 1). We then discuss the functional and ecological implications of these patterns and place our results into the broader understanding of trade-offs as constraints on the phenotype and its evolution.

## Materials and methods

### Study group

Darters, comprised of about 250 species, are a major lineage of fishes in the southeastern United States (Near et al. 2011), often co-occurring in species-rich assemblages (Carlson et al. 2009). Darters either lack or have highly reduced swim bladders (Evans and Page 2003), restricting them to rocky and sandy benthic microhabitats (Chipps et al. 1994; Stauffer et al. 1996). *Etheostoma* means “many mouths” (Rafinesque 1918), likely in reference to the variety of jaw morphologies exhibited by darters. Several distinct ecomorphs have been delineated, including species with long, pointed jaws (e.g., *Percina squamata, P. phoxocephala*, and *E. sagitta*), species with a bulbous snout (e.g., *P. caprodes, P. kathae*, and *P. austroperca*), and species with small, compact jaws (e.g., *E. simoterum, E. barrenense*, and *E. rafinesquei*; Carlson and Wainwright 2010). The remaining members of North American Percidae include perches and pikeperches (e.g., *Perca* and *Sander*; Song et al. 1998; Sloss et al. 2004), which are often larger-bodied and predatory (Mittelbach and Persson 1998; Sheppard et al. 2015).

### Morphological traits

**Fig. 2.**
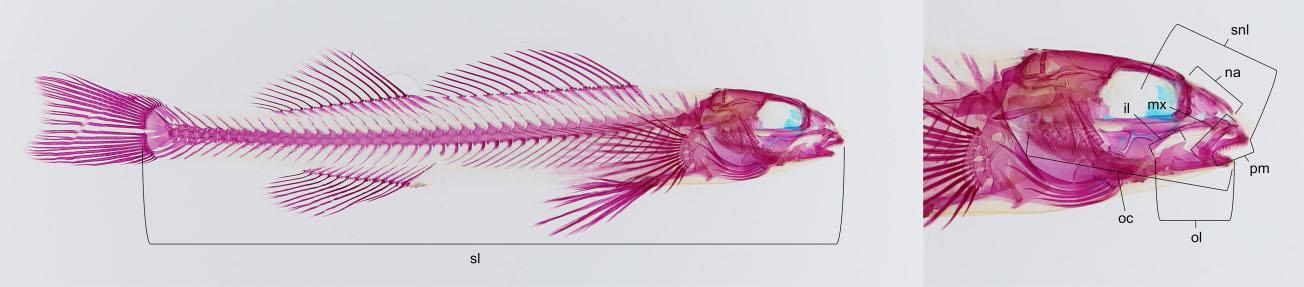
Measurements used in the study: standard length (sl), snout length (snl), oral cavity (oc), in-lever (il), out-lever (ol), maxilla (mx), premaxilla (pm), and nasal (na). Pictured is the Texas Logperch (*Percina carbonaria*; UAIC 13507.07); cleared, stained, and photographed by M.I.S.

We cleared and stained 303 specimens, representing 89 species of percid fishes from the University of Alabama Ichthyological Collection (Supplementary Table S1). Specimens were then photographed in lateral view. All measurements are linear distances measured digitally with tpsDIG2 (version 2.31; Rohlf 2017). As a proxy for the velocity–force trade-off, we used mechanical advantage (MA) of the lower jaw (Burress and Muñoz 2023), calculated as the ratio between the in- and out-levers of the lower jaw (Westneat 1994; Wainwright and Richard 1995; 2004). We chose MA because it is a simple lever that can be readily and accurately measured (but many other viable options exist—other levers, kinematic transmission, etc.; Westneat 1994; Martinez and Wainwright 2019). To characterize feeding-related morphology of the head and jaws, we measured five additional traits that characterize their shape, which is known to relate to feeding performance and/or feeding ecology (Fig. 2; Westneat 1994; Wainwright and Richard 1995; Wainwright 2007; Carlson and Wainwright 2010  Burress et al. 2023): the dentigerous arm of the premaxilla, maxilla, nasal, oral cavity length, and snout length (as well as mandible length; the out-lever). The out-lever/mandible length was measured from the anterior tip of the lower jaw to its joint with the quadrate. The in-lever was measured from the joint with the quadrate to the tip of the ascending process of the angular–articular. The premaxilla was measured from the anterior tip of the upper jaw to the posterior tip of the dentigerous arm. The maxilla was measured by taking its longest linear axis. The nasal was measured as the longest axis of the nasal bone. The oral cavity was measured from the anterior tip of the lower jaw to the posterior edge of the most posterior gill arch (Carlson and Wainwright 2010). Snout length was measured as the distance from the center of the orbit to the anterior tip of the upper jaw (Burress et al. 2023). Lastly, since these traits are expected to scale strongly with body size, we also measured standard length, measured from the anterior tip of the upper jaw to the posterior edge of the hypural plate (Fig. 2). We accounted for variation in body size by calculating the residuals from regressing each ln-transformed trait against ln-transformed standard body length using the phyl.resid function in PHYTOOLS (Revell 2012).

### Phylogenetic comparative methods

For phylogenetic comparative methods, we used the phylogeny from Rabosky et al. (2018), pruned to the 89 species in our dataset. To estimate and visualize the evolutionary history of the velocity–force trade-off among percid fishes, we used maximum likelihood, calculated with the contMap function in PHYTOOLS (Revell 2012). To evaluate whether the velocity–force trade-off constrained the phenotype, we tested for correlations between MA and craniofacial shape. Since most traits were correlated, we used principal components (PCs) to represent uncorrelated dimensions of shape. PCs were generated with the prcomp function in R version 4.1.2 (R Core Team 2022). Statistical significance was assessed with phylogenetic generalized least squares (Revell 2010). We then calculated phenotypic disparity along the extremes of the velocity–force trade-off. During this procedure, we used the phylogenetic residuals as input and disparity was calculated as variance with the morpho.disparity function in geomorph (Adams and Otárola‐Castillo 2013; Baken et al. 2021). Since the trade-off (here represented by MA) is continuous and there is no objective point at which a jaw becomes specialized for the transmission of velocity or force, we used a gradient of cut-offs to deem a jaw as velocity- or force-modified (i.e., specialized): the 10th, 15th, 17.5th, 22.5th, and 33rd percentiles along each extreme (for the lowest and highest values of MA).

Lastly, we estimated the rate of phenotypic evolution across the velocity–force trade-off using a Bayesian state-dependent model of multivariate evolution (MuSSCRat; May and Moore 2020) employed in RevBayes version 1.1.1 (Höhna et al. 2016). Size-corrected craniofacial traits were used as response variables in a multivariate framework. As the independent variable, we discretized the velocity–force trade-off using the aforementioned range of cut-offs (following Burress and Muñoz 2023; Burress and Hart 2024) and repeated analyses with each cut-off. The MuSSCRat model simultaneously estimates the evolutionary history of the continuous and discrete characters and accounts for background rate variation attributable to other, nonobserved/measured factors to reduce risk of type I error (Burress et al. 2020; May and Moore 2020; Corn et al. 2021, 2022). Additionally, we used several different priors for the number of rate shifts for each model (30, 40, and 50 rate shifts) to assess robustness to priors. Each model was run for 500k generations (after evaluating convergence across several trial runs with different numbers of generations) and assessed for effective sample size with tracer version 1.7 (Rambaut et al. 2018).

## Results

**Fig. 3.**
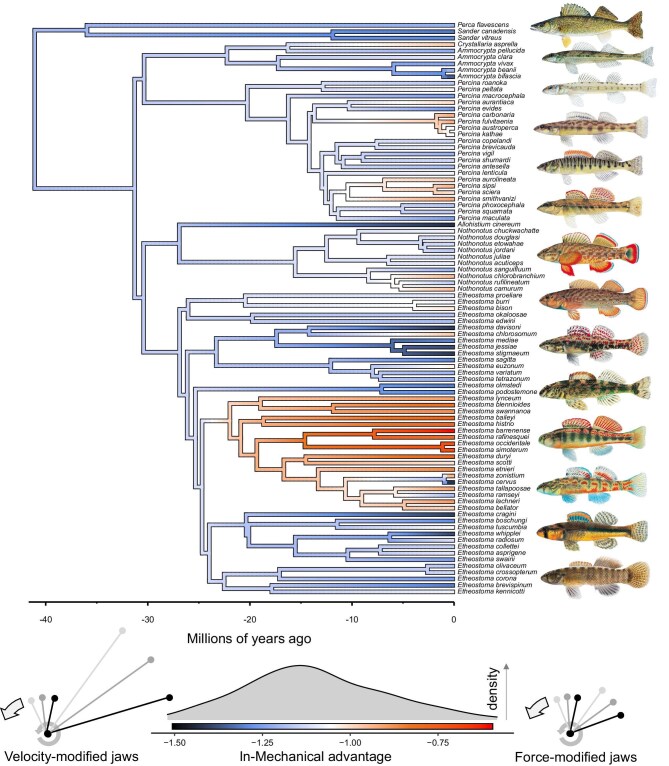
Evolutionary history of the velocity–force trade-off (here represented by mechanical advantage) among percid fishes. Plot generated with the contMap function in PHYTOOLS (Revell 2012). Illustrations courtesy of Joseph Tomelleri, used with permission, which depict an adjacent species.

**Fig. 4.**
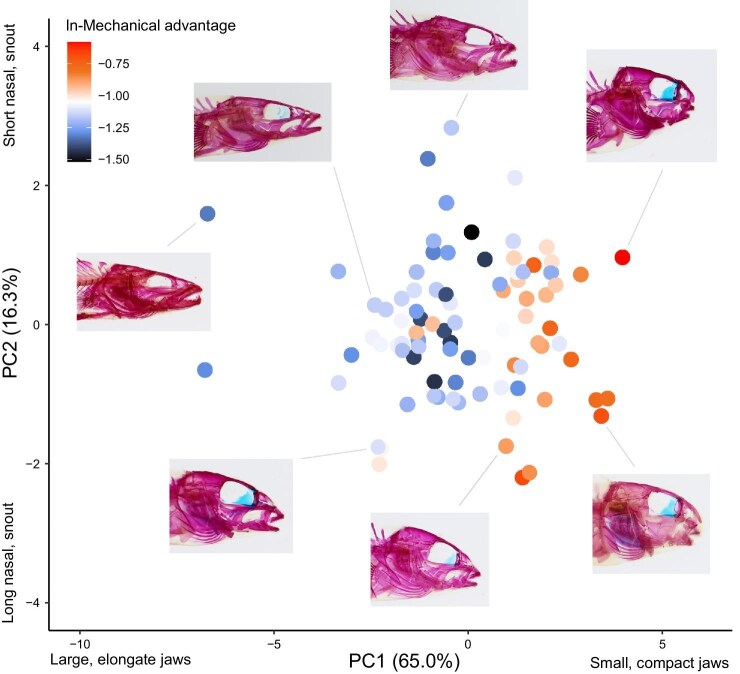
Craniofacial diversity among percid fishes. Each dot represents a species. Images depict the phenotype of the identified species. Dots are color coded based on their mechanical advantage (reflecting their position along the velocity–force trade-off; Burress & Muñoz 2023). Note that the axes are not plotted on the same scale to enhance visibility; therefore, the plot does not depict the relative variance of the two axes.

MA of percid fish jaws ranged approximately three-fold (from 0.218 to 0.559). Velocity-modified jaws arose early in the evolutionary history of percid fishes (e.g., *Perca* and *Sander*), but persisted in many derived lineages (e.g., some *Etheostoma*; Fig. 3). By contrast, highly force-modified jaws were largely confined to a single subclade of *Etheostoma*, with other independent origins of moderately force-modified jaws within *Percina* and *Nothonotus* (Fig. 3). Major axes of craniofacial diversity were the relative length of the mandible, snout, and oral cavity (Fig. 4). Some of the more extreme phenotypes (i.e., occupying the periphery of morphospace) were represented by *Sander canadensis* (large and elongate jaws), *Percina squamata* (pointed snout with long jaws), *Etheostoma kennicotti* (intermediate nasal and snout, and blunt jaws), *E. barrenense* (short nasal and small, compact jaws), and *E. blennioides* (long nasal and small, compact jaws; Fig. 4).

**Fig. 5.**
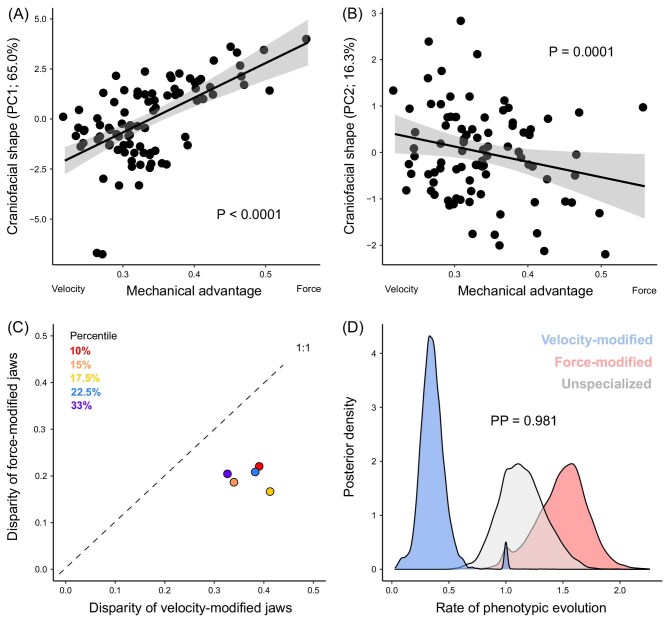
The velocity–force trade-off as an asymmetrical constraint on the phenotype. Craniofacial shape (PC1, PC2) as a function of the velocity–force trade-off (represented by mechanical advantage; **A, B**). Each point represents a species. The gray area represents the 95% confidence interval for the slope of the best fit line. Statistical significance was assessed with phylogenetic generalized least squares (Revell 2010). The relative jaw shape disparity among species that fall within various percentiles along the extremes of the velocity–force trade-off (**C**). If jaw shapes were similarly constrained along both extremes, the points would fall along the 1:1 line. Relative rates of phenotypic evolution among velocity- and force-modified jaws compared to unspecialized jaws (**D**). Statistical significance is depicted by the posterior probability (PP) that the model was state-dependent (i.e., favored over a single-rate model; see Table 1 for results from replicate models).

MA comprehensively predicted the major axes of craniofacial diversity (Fig. 5A and B), as PC1, 2, 3, 4, and 6 were significantly correlated with MA (*P* < 0.05); only PC5 was uncorrelated (*P* > 0.05). Jaws specialized for the transmission of velocity had higher phenotypic disparity than those specialized to transmit force, regardless of the cut-off used to delineate whether the jaws were specialized (all *P* < 0.05; Fig. 5C). Velocity-modified jaws exhibited a two- to three-fold slower rate of phenotypic evolution than unspecialized jaws (Fig. 5D). Force-modified jaws had a 2.5- to 4.5-fold faster rate of phenotypic evolution than velocity-modified jaws [posterior probability (PP) = 98.1; Fig. 5D]. This difference in evolutionary rate was consistent using models with different priors on the number of rate shifts and different cut-offs to delineate velocity- and force-modified jaws (all models PP > 0.78; Table 1). Although the species with the most force-modified jaws occur in a single subclade and likely drive elevated rates in the 10th and 15th percentile cut-offs, many other species with force-modified jaws occur across the phylogeny (Fig. 3). Since the method we employed to estimate the state-dependent rates of evolution accommodates background rate variation and subsequently reduces the risk of false positives (May and Moore 2020; Burress and Muñoz 2022), the elevated rates observed using more lax cut-offs (17th, 22.5th, and 33rd percentiles) are not driven by fast rates in a subset of species within the character state.

**Table 1.** Summary statistics for Bayesian state-dependent models of continuous character evolution^a^

Model	PP	RR (force:velocity)	RR (velocity:unsp)	RR (force:unsp)
10th	0.92	2.74	0.39	1.06
15th	0.86	2.22	0.59	1.31
17.5th	0.81	2.37	0.57	1.35
22.5th	0.91	3.41	0.35	1.21
33rd	0.98	4.51	0.28	1.29

^a^Posterior probability (PP) that evolutionary rate is state-dependent. Rate ratios (RR) between velocity-modified, force-modified, and unspecialized (unsp) jaws. Values are means across replicates with priors of 20, 40, and 60 rate shifts (all replicates PP > 0.78).

## Discussion

Trade-offs are widespread and ensnare a wide range of features including anatomy, life history, immunity, and other biological processes (Reznick 1983; Stearns 1989; Adamo et al. 2001; Zera and Harshman 2001; Uicker et al. 2003; Sadras 2007; Schwenke et al. 2016; Koch and Hill 2018). Despite their apparent ubiquity, the macroevolutionary implications of trade-offs are more poorly understood. The velocity–force trade-off inherent to all vertebrate jaw systems imposed a strong constraint on diversity of craniofacial morphology in percid fishes. Most notably, this constraint was asymmetrical. Velocity-modified jaws were more phenotypically diverse, yet arose more slowly over a prolonged period of evolutionary time. By contrast, force-modified jaws were more phenotypically similar and arose rapidly over shallower timescales. Highly velocity-modified jaws were also more iteratively explored during the evolutionary history of percid fishes, whereas highly force-modified jaws were largely confined to a single sublineage; although the total number of transitions toward both specializations were similar (Fig. 3). Traits involved in trade-offs may evolve rapidly (Patek et al. 2007; Holzman et al. 2012; Muñoz et al. 2017, 2018), bias phenotypic diversity (Stayton et al. 2018), and scale depending on the extent of specialization (Burress and Muñoz 2023). These patterns may underlie the broader phenomena in which phenotypic diversity and evolutionary rate vary in response to feeding ecology (Borstein et al. 2019; Arbour et al. 2020; Burress et al. 2020; Corn et al. 2021, 2022; Burress et al. 2023). The layered extent of asymmetries apparent across the velocity–force trade-off in percid fish jaws suggests that its macroevolutionary consequences could be multifunctional and widespread.

### Specialization as a constraint on the phenotype

Trade-offs, whether they are mechanical, ecological, or immunological in nature, inherently act as a constraint on the biology of organisms (Reznick 1983; Stearns 1989; Adamo et al. 2001; Zera and Harshman 2001; Uicker et al. 2003; Sadras 2007; Schwenke et al. 2016; Koch and Hill 2018). In the case of mechanical trade-offs such as the lever systems that are ubiquitous components of vertebrate anatomy, this constraint is a trade-off between the transmission of velocity versus force (Biewener 1989; Westneat 1994, 2004; Patek et al. 2007; Wainwright 2007). Force-modified jaws are associated with a biting feeding mode and crushing shelled prey. For example, rodents and birds that eat hard nuts and seeds require a forceful bite, whereas soft-bodied prey such as insects is more efficiently consumed via many, faster biting motions (Herrel et al. 2009; Zelditch et al. 2017; Navalón et al. 2019; Missagia et al. 2021). In fishes, prey capture and processing are decoupled such that the oral jaws generate suction necessary to capture prey, but tasks related to processing prey are performed by the pharyngeal jaws (Liem 1973; Wainwright et al. 2012; Burress et al. 2020). Therefore, the velocity–force trade-off in the mandible is expected to principally constrain prey capture, rather than processing; however, the two are not entirely independent (Burress and Muñoz 2021; Conith and Albertson 2021). For example, if a fish grazes snails from rock surfaces and then crushes their shells prior to ingestion, both prey capture and processing require somewhat force-modified jaws, despite the fact that two different sets of jaws perform the tasks. In other words, prey capture and processing are functionally decoupled (Liem 1973), but ecologically coupled (Burress and Muñoz 2021).

In percid fishes, we found that craniofacial shape was constrained by specialization along the velocity–force trade-off. Species with velocity-modified mandibles have large jaws, long snouts, and a large mouth cavity, whereas species with force-modified mandibles have small, compact jaws, short shouts, and a small mouth cavity (Fig. 4). These phenotypes should facilitate feeding on large, evasive prey and small attached prey, respectively (Winemiller et al. 1995). Subsequently, prey evasiveness and hardness should underly much of the dietary diversity in percids, like other ray-finned fishes (McGee et al. 2016; Martinez et al. 2018). These patterns suggest that unspecialized jaws are more variable, perhaps resulting in more opportunistic feeding strategies. Divergence in feeding ecology appears to be an important facet of percid diversity, as there are several distinct ecomorphs associated with unique feeding behaviors (Carlson and Wainwright 2010). We found that percids may have iteratively evolved velocity-modified jaws over a long timescale, as most genera contain some species with highly velocity-modified jaws; however, force-modified jaws iteratively evolved over shorter timescales, with the most specialized confined to a single subclade within *Etheostoma* (Fig. 3). Therefore, the extent to which feeding ecology drove the phenotypic diversity of percid fishes, including this apparent asymmetry, remains poorly understood.

### Asymmetrical macroevolutionary implications of trade-offs

In percid fishes the ascending process of the premaxilla is fixed or otherwise has a limited capacity to slide along the nasal bone (Carlson and Wainwright 2010), resulting in a limited ability to protrude their upper jaws, as is common among most spiny-rayed fishes (Bellwood et al. 2015). In this sense, lower jaw rotation should play a central role in the generation of suction during a feeding strike (Corn et al. 2021), and subsequently MA of the lower jaw may act as a significant constraint on craniofacial diversity. We observed that the phenotypic disparity was strongly predicted by MA and that specialization along the extremes results in reduced phenotypic diversity (Fig. 5A and B). In other words, the diversity of craniofacial morphology was strongly constrained by the velocity–force trade-off. This result is similar to that observed in cichlid fishes in which fewer jaw shapes are observed along the extremes of the velocity–force trade-off (Burress and Muñoz 2023). Further, we found that velocity-modified jaws exhibit slow rates of jaw evolution, whereas force-modified jaws exhibit accelerated rates of jaw evolution (Fig. 5D). This outcome contrasts with other spiny-rayed fishes. For example, in cichlid fishes, specialized jaws along both extremes have accelerated rates of jaw evolution relative to unspecialized jaws (Burress et al. 2020; Burress and Muñoz 2023). This discrepancy is likely attributable to the poorly protrusible jaws of percid fishes, which disproportionately affects their ability to generate suction and ultimately the jaw's ability to diversify to exploit evasive prey.

Velocity-modified jaws are associated with the generation of extreme jaw protrusion (Westneat 1994; Bellwood et al. 2015), necessary for the generation of suction to draw evasive prey into the mouth via an area of low pressure (Waltzek and Wainwright 2003; Wainwright et al. 2007). In contrast, fishes that utilize a biting mode of feeding rely more on other mechanisms such as lower jaw rotation (Martinez et al. 2018; Corn et al. 2021, 2022). Given that percid fishes have a limited ability to protrude their jaws (Carlson and Wainwright 2010), in combination, our results suggest that percid fishes exhibit limited diversification of their jaws in terms of the generation of suction, but exhibit significant diversification in terms of biting modes of feeding (Fig. 5C). This result is consistent with percid fishes mostly consuming prey with a limited capacity to evade capture such as larval insects and snails (Cordes and Page 1980; Paine et al. 1982; Van Snik Gray et al. 1997), with only a handful of species consuming highly evasive prey such as other fishes (e.g., *Perca* and *Sander*; Keast 1977; Hartman and Margraf 1992; Sheppard et al. 2015). Despite these macroevolutionary differences among fish lineages, a common theme is an emergent asymmetry such that force-modified jaws evolve faster than velocity-modified jaws (Fig. 5; Burress and Muñoz 2023).

Recent research suggests that the macroevolutionary implications of trade-offs may be asymmetrical; however, it is unclear whether this is an idiosyncrasy or a general phenomenon. Since MA is a ratio (Westneat 1994), mechanically its implications are uniform (i.e., any change in the in- and out-levers has the same impact on MA of the lever). In other words, mechanically, specialization of the lever to transmit more velocity or force should result in a uniform (or perhaps symmetrical) impact on its output (Westneat 1994; Wainwright and Richard 1995; Uicker et al. 2003, 2004). Traits involved in trade-offs tend to evolve faster than other traits (Holzman et al. 2012; Muñoz et al. 2017, 2018; Muñoz 2019). While this may explain why specialization influences the rate of evolution, it may also explain asymmetry. For example, it may be that force-modified jaws are ensnared in additional functional or ecological trade-offs. For example, in fishes, jaw systems are known to evolve at different rates in response to feeding modes or diet (Borstein et al. 2019; Burress et al. 2020; Corn et al. 2021, 2022; Burress et al. 2023). Therefore, the velocity–force trade-off may characterize a single axis of a multidimensional feeding process such that additional factors may further promote or constrain the evolution of the head and jaws. One potential such factor is the emergence of coral reefs disproportionately promoting the evolution of biting as a feeding mode among marine fishes (Corn et al. 2022). Other potential, but unknown, factors that may obfuscate or interact with the velocity–force trade-off include specialization along the benthic–pelagic axis (Friedman et al. 2020; Burress and Hart 2024) or depth axis (Martinez et al. 2021; Miller et al. 2022), which often favors the evolution of different phenotypes. We demonstrate that the macroevolutionary implication of the velocity–force trade-off is asymmetrical in fish jaws, consistent with previous studies (Burress and Muñoz 2023), but it remains unclear whether this is an emergent feature of the trade-off itself or rather due to an accumulation of secondary factors correlated with specialization along the trade-off.

## Supplementary Material

obaf013_Supplemental_Files
