# Supplementary material for: Biomechanical Specialization Acts as an Asymmetrical Constraint on the Phenotype
Source: Integr Org Biol. 2025 Apr 7;7(1):obaf013. doi: 10.1093/iob/obaf013 (PMC12012895; doi:10.1093/iob/obaf013)
Supplement: obaf013_Supplemental_Files [file obaf013_supplemental_files.zip › manuscript_submission_IOB_revision_v2_trackchanges.rtf]

Running head: Specialization constrains the phenotype


Biomechanical specialization acts as an asymmetrical constraint on the phenotype


Key words: Anatomy, Biomechanics; Diversification; Functional morphology; Macroevolution; Velocity-force trade-off


Synopsis
Vertebrate jaws involve tradeoffs between the transmission of velocity and force, which underlies their feeding performance and potentially their evolution.We investigate the velocity-force tradeoff and its implications for adaptation of the anatomically complex fish jaw system among 89 species of percid fishes (Percidae). We test alternative hypotheses about how the trade-off may symmetrically or asymmetrically constrain jaw diversity. We find that the trade-offhas a strong impact on the structural diversity of the jaws, indicating that specialization acts as a constraint on the phenotype. Force-modified jaws are compact with short snouts and a small oral cavity, while velocity-modified jaws are more robust with elongate snouts and a large oral cavity. The distribution of craniofacial diversity along the extremes is asymmetrical, as species with velocity-modified jaws are more phenotypically dissimilar than those with force-modified jaws. The rate of phenotypic evolution is also asymmetrical, as lineages with velocity- and force-modified jaws evolve slower and faster than unspecialized jaws, respectively. This discrepancy between phenotypic diversity and rate of evolution is explained by time to evolve, as force-modified jaws arose comparatively nearer the present. We expand recent literature linking trade-offs to asymmetrical macroevolutionary patterns, which may be an underappreciated cause of the uneven distribution of vertebrate diversity. 

Introduction
Trade-offs inherently impose a constraint on anatomy, life history, immunity, or other biological processes (Reznick 1983; Stearns 1989; Adamo et al. 2001; Zera and Harshman 2001; Uicker et al. 2003; Sadras 2007; Schwenke et al. 2016; Koch and Hill 2018). For example, the lever systems imbedded throughout much of vertebrate anatomy (e.g., limbs and jaws) are subject to a trade-off between the transmission of velocity versus force (Biewener 1989; Westneat 1994; 2004; Wainwright 2007; Patek et al. 2007; Zelditch et al. 2017). In addition to these mechanical properties, such trade-offs also have a broad influence on emergent functional and ecological properties, including locomotor and feeding performance as well as habitat and prey preferences (Losos 1990; Losos et al. 1993; Wainwright et al. 2004; Corn et al. 2021). 
The velocity-force trade-off is an inherent property of vertebrate jaw systems such that the jaws cannot be modified to transmit more velocity and force (Westneat 1994; 2004; Wainwright 2007; Wainwright et al. 2007). However, it is less clear is how thistrade-off might influence surrounding anatomical structures such as the craniofacial system. Many different phenotypes may produce the same mechanical properties (Wainwright et al. 2005) and many groups with exceptional phenotypic diversity have thoroughlyexplored the velocity-force trade-off (Wainwright et al. 2004; Wainwright 2007). Many macroevolutionary studies have focused on emergent properties of trade-offs (e.g., diet or feeding modes) rather than the underlying trade-off itself, and have broadly pointed to feeding ecology having a strong effect on rates of jaw evolution (Borstein et al. 2019; Arbour et al. 2020; Corn et al. 2021; 2022;  et al. 2023). Similarly, the velocity-force trade-off can strongly influence the rate of evolution (Holzman et al. 2012;  et al. 2020;  and Muñoz 2023). 
Possible macroevolutionary implications of trade-offs include funneling lineages into a limited number of possible phenotypes (Cooper and Westneat 2009;  and Muñoz 2023) and/or acting as a catalyst/constraint on rates of phenotypic evolution (Holzman et al. 2012;  et al. 2023;  and Muñoz 2023). Traits engaged in trade-offs may evolve rapidly (Holzman et al. 2012) and specialization along the extremes of a trade-off may promote rapid phenotypic evolution ( and Muñoz 2023), despite that the underlying trade-off inherently constrains the phenotype (Westneat 1994; 2004). Thus, trade-offs may have complex, often opposing, mechanical and macroevolutionary implications. 
Fish jaws are structurally complex and comprised of many lever systems (Westneat 1994) that underlie their functionality and ultimately, their emergent feeding ecology (Martinez et al. 2018;  et al. 2023). Previous work found that specialization along the extremes of the velocity-force trade-off led to rapid evolution of the surrounding jaw system in cichlid fishes, despite acting as a constraint on jaw diversity ( and Muñoz 2023). Cichlids have highly kinetic oral jaws (Waltzek and Wainwright 2003; Martinez et al. 2018;  et al. 2020) that are structurally and functionally reminiscent of most spiny-rayed fishes (Bellwood et al. 2015; McGee et al., 2016), but have exceptionally versatile pharyngeal jaws that may ease functional drawbacks associated with highly specialized oral jaws (i.e., Liem 1973). Therefore, it remains unclear if specialization along the extremes of the velocity-force trade-off acting as a catalyst of jaw evolution is a general feature of fish jaws or a more limited phenomenon enabled by highly mobile and modified oral and pharyngeal jaws, respectively. 
In many darters and their allies (Percidae), the premaxilla is fixed such that the ascending process does not slide along the nasal bone, resulting in minimal jaw protrusion (Carlson and Wainwright 2010). Since suction feeding is normally achieved via a combination of lower jaw rotation, cranial rotation, buccal depression, and jaw protrusion (Martinez et al. 2018; Corn et al. 2021), darters likely disproportionately rely upon the former three motions. Further, darters have simple pharyngeal jaws, notably lacking the modified anatomy of cichlids (Wainwright et al. 2012). Therefore, darters have comparatively less dynamic oral and pharyngeal jaw systems than cichlids and may provide further insight into how the velocity-force trade-off influences the diversity of the head and jaws. 
In this study, we assessed the role of specialization along the velocity-force trade-off as a constraint on craniofacial diversity and the rate of jaw evolution in percid fishes. We tested four possible relationships between craniofacial diversity as a function of the velocity-force trade-off: specialization (i) does not act as a constraint on the phenotype, (ii) acts as a symmetrical constraint, (iii) acts as an asymmetrical constraint in which velocity-modified jaws are disproportionately constrained, and (iv) acts as an asymmetrical constraint in which force-modified jaws are disproportionately constrained (Figure 1). We then discuss the functional and ecological implications of these patterns and place our results into the broader understanding of trade-offs as constraints on the phenotype and its evolution. 

Materials and Methods
Study group
Darters, comprised of about 250 species, are a major lineage of fishes in the southeastern United States (Near et al. 2011), often co-occurring in species rich assemblages (Carlson et al. 2009). Darters either lack or have highly reduced swim bladders (Evans and Page 2003), restricting them to rocky and sandy benthic microhabitats (Chipps et al. 1994; Stauffer et al. 1996). Etheostoma means 'many mouths' (Rafinesque 1918), likely in reference to the variety of jaw morphologies exhibited by darters. Several distinct ecomorphs have been delineated, including species with long, pointed jaws (e.g., Percina squamata, P. phoxocephala, and E. sagitta), species with a bulbous snout (e.g., P. caprodes, P. kathae, and P. austroperca), and species with small, compact jaws (e.g., E. simoterum, E. barrenense; and E. rafinesquei; Carlson and Wainwright 2010). The remaining members of North American Percidae include perches and pikeperches (e.g., Perca and Sander; Song et al. 1998; Sloss et al. 2004), which are often larger-bodied and predatory (Mittelbach and Persson 1998; Sheppard et al. 2015). 

Morphological traits
We cleared and stained 303 specimens, representing 89 species of percid fishes from the University of Alabama Ichthyological Collection (Table S1). Specimens were then photographed in lateral view. All measurements are linear distances measured digitally with tpsDIG2 (version 2.31; Rohlf 2017). As a proxy for the velocity-force trade-off, we used mechanical advantage (MA) of the lower jaw ( and Muñoz 2023), calculated as the ratio between the in- and out-levers of the lower jaw (Westneat 1994; 2004; Wainwright and Richard 1995). We chose MA because it is a simple lever that can be readily and accurately measured (but many other viable options exist – other levers, kinematic transmission, etc.; Westneat 1994; Martinez and Wainwright 2019). To characterize feeding-related morphology of the head and jaws, we measured five additional traits that characterize their shape, which is known to relate to feeding performance and/or feeding ecology (Westneat 1994; Wainwright and Richard 1995; Wainwright 2007; Carlson and Wainwright 2010  et al. 2023; Figure 2): the dentigerous arm of the premaxilla, maxilla, nasal, oral cavity length, and snout length (as well as mandible length; the out-lever). The out-lever/mandible length was measured from the anterior tip of the lower jaw to its joint with the quadrate. The in-lever was measured from the joint with the quadrate to the tip of the ascending process of the angular-articular. The premaxilla was measured from the anterior tip of the upper jaw to the posterior tip of the dentigerous arm. The maxilla was measured bytaking its longest linear axis. The nasal was measured as the longest axis of the nasal bone. The oral cavity was measured from the anterior tip of the lower jaw to the posterior edge of the most posterior gill arch (Carlson and Wainwright 2020). Snout length was measured as the distance from the center of the orbit to the anterior tip of the upper jaw ( et al. 2023). Lastly, since these traits are expected to scale strongly with body size, we also measured standard length, measured from the anterior tip of the upper jaw to the posterior edge of the hypural plate (Figure 2). We accounted for variation in body size by calculating the residuals from regressing each ln-transformed trait against ln-transformed standard body length using the phyl.resid function in PHYTOOLS (Revell 2012). 

Phylogenetic comparative methods
For phylogenetic comparative methods, we used the phylogeny from Rabosky et al. (2018), pruned to the 89 species in our dataset. To estimate and visualize the evolutionary history of the velocity-force trade-off among percid fishes, we used maximum likelihood, calculated with the contMap function in PHYTOOLS (Revell 2012). To evaluate if the velocity-force trade-off constrained the phenotype, we tested for correlations between mechanical advantage and craniofacial shape. Since most traits were correlated, we used Principal Components (PCs) to represent uncorrelated dimensions of shape. PCs were generated with the prcomp function in R version 4.1.2 (R Core Team 2022). Statistical significance was assessed with phylogenetic generalized least squares (Revell 2010). We then calculated phenotypic disparity along the extremes of the velocity-force trade-off. During this procedure, we used the phylogenetic residuals as input and disparity was calculated as variance with the morpho.disparity function in geomorph (Adams andOtárola‐Castillo 2013; Baken et al. 2021). Since the trade-off (here represented by mechanical advantage) is continuous and there is no objective point at which a jaw becomes specialized for the transmission of velocity or force, we used a gradient of cut-offs to deem a jaw as velocity- or force-modified (i.e., specialized): the 10th, 15th, 17.5th, 22.5th, and 33rd percentiles along each extreme (for the lowest and highest values of mechanical advantage). 
Lastly, we estimated the rate of phenotypic evolution across the velocity-force trade-off using a Bayesian state-dependent model of multivariate evolution (MuSSCRat; May and Moore 2020) employed in RevBayes version 1.1.1 (Höhna et al. 2016). Size-corrected craniofacial traits were used as response variables in a multivariate framework. As the independent variable, we discretized the velocity-force trade-of using the aforementioned range of cut-offs (following  and Muñoz 2023;  and Hart 2024) and repeated analyses with each cut-off. The MuSSCRat model simultaneously estimates the evolutionary history of the continuous and discrete characters and accounts for background rate variation attributable to other, non-observed/measured factors to reduce risk of type I error; May and Moore 2020;  et al. 2020; Corn et al. 2021; 2022). Additionally, we used several different priors for the number of rate shifts for each model (30, 40, and 50 rate shifts) to assess robustness to priors. Each model was run for 500k generations (after evaluating convergence across several trial runs with different numbers of generations) and were assessed for effective sample size with tracer version 1.7 (Rambaut et al. 2018). 

Results
Mechanical advantage of percid fish jaws ranged approximately 3-fold (from 21.8 to 55.9). Velocity-modified jaws arose early in the evolutionary history of percid fishes (e.g., Perca and Sander), but persisted in many derived lineages (e.g., some Etheostoma; Figure 3). By contrast, highly force-modified jaws were largely confined to a single subclade of Etheostoma, with other independent origins of moderately force-modified jaws within Percina and Nothonotus (Figure 3). Major axes of craniofacial diversity were the relative length of the mandible, snout, and oral cavity (Figure 4). Some of the more extreme phenotypes (i.e., occupying the periphery of morphospace) were represented by Sander canadensis (large and elongate jaws), Percina squamata (pointed snout with long jaws), Etheostoma kinnicotti (intermediate nasal and snout, and blunt jaws), E. barrenense (short nasal and small, compact jaws), and E. blennioides (long nasal and small, compact jaws; Figure 4). 
Mechanical advantage comprehensively predicted the major axes of craniofacial diversity (Figure 5a,b), as PC1, 2, 3, 4, and 6 were significantly correlated with mechanical advantage (P < 0.05); only PC5 was uncorrelated (P > 0.05). Jaws specialized for the transmission of velocity had higher phenotypic disparity than those specialized to transmit force, regardless of the cut-off used to delineate if the jaws were specialized (all P <0.05; Figure 5c). Velocity-modified jaws exhibited a 2 to 3-fold slower rate of phenotypic evolution than unspecialized jaws (Figure 5d). Force-modified jaws had a 2.5 to 4.5-fold faster rate of phenotypic evolution than velocity-modified jaws (Posterior probability [PP] = 98.1; Figure 5d). This difference in evolutionary rate was consistent using models with different priors on the number of rate shifts and different cut-offs to delineate velocity- and force-modified jaws (all models PP > 0.78; Table 1). Although the species with the most force-modified jaws occur in a single sub-clade and likely drive elevated rates in the 10th and 15th percentile cut-offs, many other species with force-modified jaws occur across the phylogeny (Figure 3). Since the method we employed to estimate the state-dependent rates of evolution accommodates background rate variation and subsequently reduces the risk of false positives (May and Moore 2020; Burress and Muñoz 2022), the elevated rates observed using more lax cut-offs (17th, 22.5th, and 33rd percentiles) are not driven by fast rates in a fewsubset of species within the character state.

Discussion
Trade-offs are widespread and ensnare a wide range of features including anatomy, life history, immunity, and other biological processes (Reznick 1983; Stearns 1989; Adamo et al. 2001; Zera and Harshman 2001; Uicker et al. 2003; Sadras 2007; Schwenke et al. 2016; Koch and Hill 2018). Despite their apparent ubiquity, the macroevolutionary implications of trade-offs are more poorly understood. The velocity-force trade-off inherent to all vertebrate jaw systems imposed a strong constraint on diversity of craniofacial morphology in percid fishes. Most notably, this constraint was asymmetrical. Velocity-modified jaws were more phenotypically diverse, yet arose more slowly over a prolonged period of evolutionary time. By contrast, force-modified jaws were more phenotypically similar and arose rapidly over shallower timescales. Highly velocity-modified jaws were also more iteratively explored during the evolutionary history of percid fishes, whereas highly force-modified jaws were largely confined to a single sub-lineage; although to total number of transitions toward both specializations were similar (Figure3). Traits involved in trade-offs may evolve rapidly (Patek et al. 2007; Holzman et al. 2012; Muñoz et al. 2017; 2018), bias phenotypic diversity (Stayton et al. 2018), and scale depending on the extent of specialization ( and Muñoz 2023). These patterns may underlie the broader phenomena in which phenotypic diversity and evolutionary rate vary in response to feeding ecology (Borstein et al. 2019; Arbour et al. 2020; Corn et al. 2021; 2022;  et al. 2020; 2023). The layered extent of asymmetries apparent across the velocity-force trade-off in percid fish jaws suggests its macroevolutionary consequences could be multifunctional and widespread. 

Specialization as a constraint on the phenotype
Trade-offs, whether they are mechanical, ecological, or immunological in nature, inherently act as a constraint on the biology of organisms (Reznick 1983; Stearns 1989; Adamo et al. 2001; Zera and Harshman 2001; Uicker et al. 2003; Sadras 2007; Schwenke et al. 2016; Koch and Hill 2018). In the case of mechanical trade-offs such as the lever systems that are ubiquitous components of vertebrate anatomy, this constraint is trade-off between the transmission of velocity versus force (Biewener 1989; Westneat 1994; 2004; Wainwright 2007; Patek et al. 2007). Force-modified jaws are associated with a biting feeding mode and crushing shelled prey. For example, rodents and birds that eat hard nuts and seeds require a forceful bite, whereas soft-bodied prey like insects is more efficiently consumed via many, faster biting motions (Herrel et al. 2009; Zelditch et al. 2017; Missagia et al. 2021; Navalón et al. 2020). In fishes, prey capture and processing are decoupled such that the oral jaws generate suction necessary to capture prey, but tasks related to processing prey are performed by the pharyngeal jaws (Liem 1973; Wainwright et al. 2012;  et al. 2020). Therefore, the velocity-force trade-off in the mandible is expected to principally constrain prey capture rather processing; however, the two are not entirely independent ( and Muñoz 2021; Conith and Albertson 2021). For example, if a fish grazes snails from rock surfaces and then crushes their shells prior to ingestion, both prey capture and processing require somewhat force-modified jaws, despite that two different sets of jaws perform the tasks. In other words, prey capture and processing are functionally decoupled (Liem 1973), but ecologically coupled ( and Muñoz 2021).
	In percid fishes, we found that craniofacial shape  was constrained by specialization along the velocity-force trade-off. Species with velocity-modified mandibles have large jaws, long snouts, and a large mouth cavity, whereas species with force-modified mandibles have small, compact jaws, short shouts, and a small mouth cavity (Figure 4). These phenotypes should facilitate feeding on large, evasive prey and small attached prey, respectively (Winemiller et al. 1995). Subsequently, prey evasiveness and hardness should underly much of the dietary diversity in percids, like other ray-finned fishes (McGee et al. 2016; Martinez et al. 2018).These patterns suggest that unspecialized jaws are more variable, perhaps resulting in more opportunistic feeding strategies. Divergence in feeding ecology appears to be an important facet of percid diversity, as there are several distinct ecomorphs associated with unique feeding behaviors (Carlson and Wainwright 2010). We found that percids may have iteratively evolved velocity-modified jaws over a long timescale, as most genera contain some species with highly velocity-modified jaws; however, force-modified jaws iteratively evolved over shorter timescales, with the most specialized confined to a single sub-clade within Etheostoma (Figure 3). Therefore, the extent to which feeding ecology drove the phenotypic diversity of percid fishes, including this apparent asymmetry, remains poorly understood.

Asymmetrical macroevolutionary implications of trade-offs 
In percid fishes the ascending process of the premaxilla is fixed or otherwise has a limited capacity to slide along the nasal bone (Carlson and Wainwright 2010), resulting in a limited ability to protrude their upper jaws, as is common among most spiny-rayed fishes (Bellwood et al. 2015). In this sense, lower jaw rotation should play a central role in the generation of suction during a feeding strike (Corn et al. 2021), and subsequently mechanical advantage of the lower jaw may act as a significant constraint on craniofacial diversity. We observed that the phenotypic disparity was strongly predicted by mechanical advantage and that specialization along the extremes result in reduced phenotypic diversity (Figure 5a,b). In other words, the diversity of craniofacial morphology was strongly constrained by the velocity-force trade-off. This result is similar to that observed in cichlid fishes in which fewer jaw shapes are observed along the extremes of the velocity-force trade-off ( and Muñoz 2023). Further, we found that velocity-modified jaws exhibit slow rates of jaw evolution, whereas force-modified jaws exhibit accelerated rates of jaw evolution (Figure 5d). This outcome contrasts with other spiny-rayed fishes. For example, in cichlid fishes, specialized jaws along both extremes have accelerated rates of jaw evolution relative to unspecialized jaws ( et al. 2020;  and Muñoz 2023). This discrepancy is likely attributable to the poorly protrusible jaws of percid fishes, which disproportionately affects their ability to generate suction and ultimately the jaw's ability to diversify to exploit evasive prey. 
Velocity-modified jaws are associated with the generation of extreme jaw protrusion (Westneat 1994; Bellwood et al. 2015), necessary for the generation of suction to draw evasive prey into the mouth via an area of low pressure (Waltzek and Wainwright 2003; Wainwright et al. 2007). In contrast, fishes that utilize a biting mode of feeding rely more on other mechanisms such as lower jaw rotation (Martinez et al. 2018; Corn et al. 2021; 2022). Given that percid fishes have a limited ability to protrude their jaws (Carlson and Wainwright 2010), in combination, our results suggest that percid fishes exhibit limited diversification of their jaws in terms of the generation of suction, but exhibit significant diversification in terms of biting modes of feeding (Figure 5c). This result is consistent with percid fishes mostly consuming prey with a limited capacity to evade capture such as larval insects and snails (Cordes and Page 1980; Paine et al. 1982; Van Snik et al. 1997), with only a handful of species consuming highly evasive prey such as other fishes (e.g., Perca and Sander; Keast 1977; Hartman and Margraf 1992; Sheppard et al. 2015). Despite these macroevolutionary differences among fish lineages, a common theme is an emergent asymmetry such that force-modified jaws evolve faster than velocity-modified jaws (Figure 5;  and Muñoz 2023). 
	Recent research suggests that the macroevolutionary implications of trade-offs may be asymmetrical; however, it is unclear if this is an idiosyncrasy or a general phenomenon. Since mechanical advantage is a ratio (Westneat 1994), mechanically its implications are uniform (i.e., any change in the in- and out-levers have the same impact on mechanical advantage of the lever). In other words, mechanically, specialization of the lever to transmit more velocity or force should result in a uniform (or perhaps symmetrical) impact on its output (Westneat 1994; 2004; Wainwright and Richard 1995; Uicker et al. 2003). Traits involved in trade-offs tend to evolve faster than other traits (Holzman et al. 2012; Muñoz et al. 2017; 2018; Muñoz 2019). While this may explain why specialization influences the rate of evolution, it may also explain asymmetry. For example, it may be that force-modified jaws are ensnared in additional function or ecological trade-offs. For example, in fishes, jaw systems are known to evolve at different rates in response to feeding modes or diet (Borstein et al. 2019;  et al. 2020; 2023; Corn et al. 2021; 2022). Therefore, the velocity-force trade-off may characterize a single axis of a multidimensional feeding process such that additional factors may further promote or constrain the evolution of the head and jaws. One potential such factor is the emergence of coral reefs disproportionately promoting the evolution of biting as a feeding mode among marine fishes (Corn et al. 2022). Other potential, but unknown, factors that may obfuscate or interact with the velocity-force trade-off include specialization along the benthic-pelagic axis (Friedman et al. 2020;  and Hart 2024) or depth axis (Martinez et al. 2021; Miller et al. 2022), which often favors the evolution of different phenotypes. We demonstrate that the macroevolutionary implications of the velocity-force trade-off is asymmetrical in fish jaws, consistent with previous studies ( and Muñoz 2023), but it remains unclear if this is an emergent feature of the trade-off itself or rather due to an accumulation of secondary factors correlated with specialization along the trade-off. 

Conflict of Interest
The authors declare no conflict of interest.

References
Adamo, S.A., Jensen, M., & Younger, M. (2001). Changes in lifetime immunocompetence in male and female Gryllus texensis (formerly G. integer): trade-offs between immunity and reproduction. Animal Behaviour, 62(3), 417-425.
Adams, D.C., & Otárola‐Castillo, E. (2013). geomorph: an R package for the collection and analysis of geometric morphometric shape data. Methods in ecology and evolution, 4(4), 393-399.
Arbour, J.H., Montaña, C.G., Winemiller, K.O., Pease, A.A., Soria-Barreto, M., Cochran-Biederman, J.L., & López-Fernández, H. (2020). Macroevolutionary analyses indicate that repeated adaptive shifts towards predatory diets affect functional diversity in Neotropical cichlids. Biological Journal of the Linnean Society, 129(4), 844-861.
Baken, E.K., Collyer, M.L., Kaliontzopoulou, A., & Adams, D.C. (2021). geomorph v4. 0 and gmShiny: Enhanced analytics and a new graphical interface for a comprehensive morphometric experience. Methods in Ecology and Evolution, 12(12), 2355-2363.
Bellwood, D.R., Goatley, C.H., Bellwood, O., Delbarre, D.J., & Friedman, M. (2015). The rise of jaw protrusion in spiny-rayed fishes closes the gap on elusive prey. Current Biology, 25(20), 2696-2700.
Biewener, A.A. (1989). Scaling body support in mammals: limb posture and muscle mechanics. Science, 245(4913), 45-48.
Borstein, S.R., Fordyce, J.A., O'Meara, B.C., Wainwright, P.C., & McGee, M.D. (2019). Reef fish functional traits evolve fastest at trophic extremes. Nature ecology & evolution, 3(2), 191-199.
, E.D., & Hart, P.B. (2024). Pelagic zone is an evolutionary catalyst, but an ecological dead end, for North American minnows. Evolution, p.qpae062.
, E.D., Piálek, L., Casciotta, J., Almirón, A., & Øíèan, O. (2023). Rapid parallel morphological and mechanical diversification of South American pike cichlids (Crenicichla). Systematic Biology, 72(1), 120-133.
, E.D., & Muñoz, M.M. (2023). Functional trade-offs asymmetrically promote phenotypic evolution. Systematic Biology, 72(1), 150-160.
, E.D. and Muñoz, M.M., 2022. Ecological opportunity from innovation, not islands, drove the anole lizard adaptive radiation.Systematic Biology, 71(1), 93-104.
, E.D., & Muñoz, M.M. (2021). Ecological limits on the decoupling of prey capture and processing in fishes. Integrative and Comparative Biology, 61(3), 773-782.
, E.D., Martinez, C.M., & Wainwright, P.C. (2020). Decoupled jaws promote trophic diversity in cichlid fishes. Evolution, 74(5), 950-961.
Carlson, R.L., & Wainwright, P.C. (2010). The ecological morphology of darter fishes (Percidae: Etheostomatinae). Biological Journal of the Linnean Society, 100(1), 30-45.
Carlson, R.L., Wainwright, P.C., & Near, T.J. (2009). Relationship between species co-occurrence and rate of morphological change in Percina darters (Percidae: Etheostomatinae). Evolution, 63(3), pp.767-778.
Chipps, S.R., Perry, W.B., & Perry, S.A. (1994). Patterns of microhabitat use among four species of darters in three Appalachian streams. American Midland Naturalist, 175-180.
Conith, A.J., & Albertson, R.C. (2021). The cichlid oral and pharyngeal jaws are evolutionarily and genetically coupled. Nature Communications, 12, 5477.
Cooper, W.J., & Westneat, M.W. (2009). Form and function of damselfish skulls: rapid and repeated evolution into a limited number of trophic niches. BMC evolutionary biology, 9, 1-17.
Cordes, L.E., & Page, L.M. (1980). Feeding chronology and diet composition of two darters (Percidae) in the Iroquois River system, Illinois. American Midland Naturalist, 202-206.
Corn, K.A., Friedman, S.T., , E.D., Martinez, C.M., Larouche, O., Price, S.A., & Wainwright, P.C. (2022). The rise of biting during the Cenozoic fueled reef fish body shape diversification. Proceedings of the National Academy of Sciences, 119(31), p.e2119828119.
Corn, K.A., Martinez, C.M., , E.D., Wainwright, P.C. (2021). A multifunction trade-off has contrasting effects on the evolution of form and function. Systematic Biology, 70(4), 681-693.
Evans, D.J., Page, L.M. (2003). Distribution and relative size of the swim bladder in Percina, with comparisons to Etheostoma, Crystallaria, and Ammocrypta (Teleostei: Percidae). Environmental biology of fishes, 66, 61-65.
Hartman, K.J., & Margraf, F.J. (1992). Effects of prey and predator abundances on prey consumption and growth of walleyes in western Lake Erie. Transactions of the American Fisheries Society, 121(2), 245-260.
Friedman, S.T., Price, S.A., Corn, K.A., Larouche, O., Martinez, C.M., & Wainwright, P.C. (2020). Body shape diversification along the benthic–pelagic axis in marine fishes. Proceedings of the Royal Society B, 287(1931), p.20201053.
Holzman, R., Collar, D.C., Price, S.A., Hulsey, C.D., Thomson, R.C., & Wainwright, P.C. (2012). Biomechanical trade-offs bias rates of evolution in the feeding apparatus of fishes. Proceedings of the Royal Society B: Biological Sciences, 279(1732), 1287-1292.
Höhna, S., Landis, M.J., Heath, T.A., Boussau, B., Lartillot, N., Moore, B.R., Huelsenbeck, J.P., & Ronquist, F. (2016). RevBayes: Bayesian phylogenetic inference using graphical models and an interactive model-specification language. Systematic biology, 65(4), 726-736.
Holzman, R., Collar, D.C., Price, S.A., Hulsey, C.D., Thomson, R.C., & Wainwright, P.C.(2012). Biomechanical trade-offs bias rates of evolution in the feeding apparatus of fishes. Proceedings of the Royal Society B: Biological Sciences, 279(1732), 1287-1292.
Keast, A. (1977). Diet overlaps and feeding relationships between the year classes in the yellow perch (Perca flavescens). Environmental Biology of Fishes, 2, 53-70.
Koch, R.E., & Hill, G.E. (2018). Do carotenoid‐based ornaments entail resource trade‐offs? An evaluation of theory and data. Functional Ecology, 32(8), 1908-1920.
Liem, K.F. (1973). Evolutionary strategies and morphological innovations: cichlid pharyngeal jaws. Systematic zoology, 22(4), 425-441.
Losos, J.B., Walton, B.M., & Bennett, A.F. (1993). Trade-offs between sprinting and clinging ability in Kenyan chameleons. Functional Ecology, 7(3), 281-286.
Losos, J.B. (1990). The evolution of form and function: morphology and locomotor performance in West Indian Anolis lizards. Evolution, 44(5), 1189-1203.
Martinez, C.M., Friedman, S.T., Corn, K.A., Larouche, O., Price, S.A., & Wainwright, P.C. (2021). The deep sea is a hot spot of fish body shape evolution. Ecology letters, 24(9), 1788-1799.
Martinez, C.M., & Wainwright, P.C. (2019). Extending the geometric approach for studying biomechanical motions. Integrative and Comparative Biology, 59, 684-695.
Martinez, C.M., McGee, M.D., Borstein, S.R., & Wainwright, P.C. (2018). Feeding ecology underlies the evolution of cichlid jaw mobility. Evolution, 72(8), 1645-1655.
May, M.R., & Moore, B.R. (2020). A Bayesian approach for inferring the impact of a discrete character on rates of continuous-character evolution in the presence of background-rate variation. Systematic biology, 69(3), 530-544.
McGee, M.D., Faircloth, B.C., Borstein, S.R., Zheng, J., Darrin Hulsey, C., Wainwright, P.C., & Alfaro, M.E. (2016). Replicated divergence in cichlid radiations mirrors a major vertebrate innovation. Proceedings of the Royal Society B: Biological Sciences, 283(1822), p.20151413.
Miller, E.C., Martinez, C.M., Friedman, S.T., Wainwright, P.C., Price, S.A., & Tornabene, L. (2022). Alternating regimes of shallow and deep-sea diversification explain a species-richness paradox in marine fishes. Proceedings of the National Academy of Sciences, 119(43), p.e2123544119.
Missagia, R.V., Patterson, B.D., Krentzel, D., & Perini, F.A. 2021. Insectivory leads to functional convergence in a group of Neotropical rodents. Journal of Evolutionary Biology, 34(2), 391-402.
Mittelbach, G.G., & Persson, L. (1998). The ontogeny of piscivory and its ecological consequences. Canadian Journal of Fisheries and Aquatic Sciences, 55(6), 1454-1465.
Muñoz, M.M. (2019). The evolutionary dynamics of mechanically complex systems. Integrative and Comparative Biology, 59(3), 705-715.
Muñoz, M.M., Hu, Y., Anderson, P.S., & Patek, S.N. (2018). Strong biomechanical relationships bias the tempo and mode of morphological evolution. Elife, 7, p.e37621.
Muñoz, M.M., Anderson, P.S., & Patek, S.N. (2017). Mechanical sensitivity and the dynamics of evolutionary rate shifts in biomechanical systems. Proceedings of the Royal Society B: Biological Sciences, 284(1847), p.20162325.
Near, T.J., Bossu, C.M., Bradburd, G.S., Carlson, R.L., Harrington, R.C., Hollingsworth Jr, P.R., Keck, B.P., & Etnier, D.A. (2011). Phylogeny and temporal diversification of darters (Percidae: Etheostomatinae). Systematic Biology, 60(5), 565-595.
Paine, M.D., Dodson, J.J., & Power, G. (1982). Habitat and food resource partitioning among four species of darters (Percidae: Etheostoma) in a southern Ontario stream. Canadian Journal of Zoology, 60(7), 1635-1641.
Patek, S.N., Nowroozi, B.N., Baio, J.E., Caldwell, R.L., & Summers, A.P. (2007). Linkage mechanics and power amplification of the mantis shrimp's strike. Journal of Experimental Biology, 210(20), 3677-3688.
R Core Team. (2022). R: A Language and Environment for Statistical Computing https://www. R-project. org. R Foundation for Statistical Computing.
Rabosky, D.L., Chang, J., Title, P.O., Cowman, P.F., Sallan, L., Friedman, M., Kaschner, K., Garilao, C., Near, T.J., Coll, M., & Alfaro, M.E. (2018). An inverse latitudinal gradient in speciation rate for marine fishes. Nature, 559(7714), 392-395.
Rafinesque, C.S. (1918). Prodome de 70 nouveaux genres d-animaux découverts dans l'intérieur des États-Unis d'Amérique, Durant l'année 1818. 
Rambaut, A., Drummond, A.J., Xie, D., Baele, G., & Suchard, M.A. (2018). Posterior summarization in Bayesian phylogenetics using Tracer 1.7. Systematic biology, 67(5), 901-904.
Revell, L.J. (2012). phytools: an R package for phylogenetic comparative biology (and other things). Methods in ecology and evolution, (2), 217-223.
Revell, L.J. (2010). Phylogenetic signal and linear regression on species data. Methods in Ecology and Evolution, 1(4), 319-329.
Reznick, D. (1983). The structure of guppy life histories: the tradeoff between growth and reproduction. Ecology, 64(4), 862-873.
Rohlf, F.J. (2017). tpsDig2, digitize landmarks and outlines, version 2.31. Department of Ecology and Evolution, State University of New York at Stony Brook.
Sadras, V.O. (2007). Evolutionary aspects of the trade-off between seed size and number in crops. Field Crops Research, 100(2-3), 125-138.
Sheppard, K.T., Davoren, G.K., & Hann, B.J. (2015). Diet of walleye and sauger and morphological characteristics of their prey in Lake Winnipeg. Journal of Great Lakes Research, 41(3), 907-915.
Sloss, B.L., Billington, N., & Burr, B.M. (2004). A molecular phylogeny of the Percidae (Teleostei, Perciformes) based on mitochondrial DNA sequence. Molecular Phylogenetics and Evolution, 32(2), 545-562.
Song, C.B., Near, T.J., & Page, L.M. (1998). Phylogenetic relations among percid fishes as inferred from mitochondrial cytochromebDNA sequence data. Molecular Phylogenetics and Evolution, 10(3), 343-353.
Stayton, C.T., O'Connor, L.F., & Nisivoccia, N.M. (2018). The influence of multiple functional demands on morphological diversification: a test on turtle shells. Evolution, 72(9), 1933-1949.
Stearns, S.C. (1989). Trade-offs in life-history evolution. Functional Ecology 3: 259-268.
Schwenke, R.A., Lazzaro, B.P., & Wolfner, M.F. (2016). Reproduction–immunity trade-offs in insects. Annual review of entomology, 61, 239-256.
Stauffer, J.R., Boltz, J.M., Kellogg, K.A., & Van Snik, E.S. (1996). Microhabitat partitioning in a diverse assemblage of darters in the Allegheny River system. Environmental Biology of Fishes, 46, 37-44.
Uicker, J.J., G.R. Pennock, J.E. Shigley, & J.M. McCarthy. (2003). Theory of machines and mechanisms, vol. 3. New York: Oxford University Press.
Van Snik Gray, E., Boltz, J.M., Kellogg, K.A., & Stauffer Jr, J.R. (1997). Food resource partitioning by nine sympatric darter species. Transactions of the American Fisheries Society, 126(5), 822-840.
Wainwright, P.C., Smith, W.L., Price, S.A., Tang, K.L., Sparks, J.S., Ferry, L.A., Kuhn, K.L., Eytan, R.I., & Near, T.J. (2012). The evolution of pharyngognathy: a phylogenetic and functional appraisal of the pharyngeal jaw key innovation in labroid fishes and beyond. Systematic Biology, 61(6), 1001-1027.
Wainwright, P.C. (2007). Functional versus morphological diversity in macroevolution. Annu. Rev. Ecol. Evol. Syst., 38, 381-401.
Wainwright, P., Carroll, A.M., Collar, D.C., Day, S.W., Higham, T.E., & Holzman, R.A. (2007). Suction feeding mechanics, performance, and diversity in fishes. Integrative and comparative biology, 47(1), 96-106.
Wainwright, P.C., Alfaro, M.E., Bolnick, D.I. and Hulsey, C.D., 2005. Many-to-one mapping of form to function: a general principle in organismal design? Integrative and comparative biology, 45(2), pp.256-262.
Wainwright, P.C., Bellwood, D.R., Westneat, M.W., Grubich, J.R., & Hoey, A.S. (2004). A functional morphospace for the skull of labrid fishes: patterns of diversity in a complex biomechanical system. Biological Journal of the Linnean Society, 82(1), 1-25.
Wainwright, P.C., & Richard, B.A. (1995). Predicting patterns of prey use from morphology of fishes. Environmental biology of fishes, 44, 97-113.
Waltzek, T.B., & Wainwright, P.C. (2003). Functional morphology of extreme jaw protrusion in Neotropical cichlids. Journal of Morphology, 257(1), 96-106.
Westneat, M.W. (2004). Evolution of levers and linkages in the feeding mechanisms of fishes. Integrative and Comparative Biology, 44(5), 378-389.
Westneat, M.W. (1994). Transmission of force and velocity in the feeding mechanisms of labrid fishes (Teleostei, Perciformes). Zoomorphology, 114(2), 103-118.
Winemiller, K.O., Kelso-Winemiller, L.C., & Brenkert, A.L. (1995). Ecomorphological diversification and convergence in fluvial cichlid fishes. Ecomorphology of fishes, 44, 235-261.
Zelditch, M.L., Ye, J., Mitchell, J.S., & Swiderski, D.L. (2017). Rare ecomorphological convergence on a complex adaptive landscape: body size and diet mediate evolution of jaw shape in squirrels (Sciuridae). Evolution, 71(3), 633-649.
Zera, A.J., & Harshman, L.G. (2001). The physiology of life history trade-offs in animals. Annual review of Ecology and Systematics, 32(1), 95-126.

Table 1. Summary statistics for Bayesian state-dependent models of continuous character evolution. Posterior probability (PP) that evolutionary rate is state-dependent. Rate ratios (RR) between velocity-modified, force-modified, and unspecialized (unsp) jaws. Values are means across replicates with priors of 20, 40, and 60 rate shifts (all replicates PP > 0.78).
Model	PP	RR (force:velocity)	RR (velocity:unsp)	RR (force:unsp)	
10th	0.92 	2.74	0.39	1.06	
15th	0.86	2.22	0.59	1.31	
17.5th	0.81	2.37	0.57	1.35	
22.5th	0.91 	3.41	0.35	1.21	
33rd	0.98 	4.51	0.28	1.29	


Figure Legends
Figure 1. Hypothetical phenotypic diversity as a function of the velocity-force trade-off: the trade-off does not act as a constraint on phenotypic diversity (A), acts as a symmetrical constraint on phenotypic diversity (B), acts as an asymmetrical constraint in which force-modified jaws are disproportionately constrained (C), and acts as an asymmetrical constraint in which velocity-modified jaws are disproportionately constrained (D). 

Figure 2. Measurements used in the study: standard length (sl), snout length (snl), oral cavity (oc), in-lever (il), out-lever (ol), maxilla (mx), premaxilla (pm), and nasal (na). Pictured is the Texas Logperch (Percina carbonaria; UAIC 13507.07); cleared, stained, and photographed by MIS.  

Figure 3. Evolutionary history of the velocity-force trade-off (here represented by mechanical advantage) among percid fishes. Plot generated with the contMap function in PHYTOOLS (Revell, 2012). Illustrations courtesy of Joseph Tomelleri, used with permission, which depict an adjacent species.

Figure 4. Craniofacial diversity among percid fishes. Each dot represents a species. Images depict the phenotype of the identified species. Dots are color coded based on their mechanical advantage (reflecting their position along the velocity-force trade-off;  & Muñoz, 2023). Note that the axes are not plotted on the same scale to enhance visibility, therefore, the plot does not depict the relative variance of the two axes. 

Figure 5. The velocity-force trade-off as an asymmetrical constraint on the phenotype. Craniofacial shape (PC1, PC2) as a function of the velocity-force trade-off (represented by mechanical advantage; A, B). Each point represents a species. The gray area represents the 95% confidence interval for the slope of the best fit line. Statistical significance was assessed with phylogenetic generalized least squares (Revell, 2010). The relative jaw shape disparity among species that fall within various percentiles along the extremes of the velocity-force trade-off (C). If jaw shapes were similarly constrained along both extremes, the points would fall along the 1:1 line. Relative rates of phenotypic evolution among velocity- and force-modified jaws compared to unspecialized jaws (D). Statistical significance is depicted by the posterior probability (PP) that the model was state-dependent (i.e., favored over a single rate model; see Table 1 for results from replicate models). 
